# A neural learning approach for simultaneous object detection and grasp detection in cluttered scenes

**DOI:** 10.3389/fncom.2023.1110889

**Published:** 2023-02-20

**Authors:** Yang Zhang, Lihua Xie, Yuheng Li, Yuan Li

**Affiliations:** ^1^China Tobacco Sichuan Industrial Co., Ltd, Chengdu, Sichuan, China; ^2^Qinhuangdao Tobacco Machinery Co., Ltd, Qinhuangdao, Hebei, China

**Keywords:** grasp detection, object detection, RGB-D image, deep neural network, robotic manipulation

## Abstract

Object detection and grasp detection are essential for unmanned systems working in cluttered real-world environments. Detecting grasp configurations for each object in the scene would enable reasoning manipulations. However, finding the relationships between objects and grasp configurations is still a challenging problem. To achieve this, we propose a novel neural learning approach, namely SOGD, to predict a best grasp configuration for each detected objects from an RGB-D image. The cluttered background is first filtered out via a 3D-plane-based approach. Then two separate branches are designed to detect objects and grasp candidates, respectively. The relationship between object proposals and grasp candidates are learned by an additional alignment module. A series of experiments are conducted on two public datasets (Cornell Grasp Dataset and Jacquard Dataset) and the results demonstrate the superior performance of our SOGD against SOTA methods in predicting reasonable grasp configurations “from a cluttered scene.”

## 1. Introduction

Automated object grasping is essential and challenging to robots or unmanned systems working in real-world cluttered scenarios. As a core component of autonomous grasping, grasp detection, which outputs the most possible grasp configuration for the manipulator, has attracted great attention from both academic and industrial communities. Existing methods often predict a series of possible grasp configurations based on the input images (Depierre et al., 2018; Zhang et al., 2019; Wang et al., 2021; Yu et al., 2022b). When encountered with a cluttered scene, which is a common case in our daily life, we humans often identify the target object first and then determine the best pose to grab the object. This provides two kinds of benefits: (1) we can easily explain why the predicted grasp configuration is the best, and (2) our efforts will be focused on the object area instead of the cluttered background to make a better decision. However, most previous studies do not have a strong ability to model the relationship between the target objects and the predicted grasp configurations. In order to make grasp detection more accurate and reasonable, we investigate the problem of simultaneous object detection and grasp detection, where the best grasp configuration is predicted for each detected object in the cluttered scene.

Since object manipulation is performed in a 3D space, using a 3D representation for grasp detection is a natural way. A grasp candidate is a 6-DOF gripper pose *g* = (*x, y, z, r*_*x*_, *r*_*y*_, *r*_*z*_), *g*∈**SE**(3), with the 3D position and rotation angles along each axis of the gripper. Methods based on this 3D representation (Pas et al., 2017; Liang et al., 2019) often generate a large number of candidates and then evaluate whether it is a good grasp according to a specific criterion. These methods are easy to understand but often suffer efficiency problems due to 3D operations. Motivated by the superior performance of deep learning technology on detection or segmentation tasks (Cheon et al., 2022; Huang et al., 2022; Khan et al., 2022), image-based deep models have become popular for grasp detection (Chu et al., 2018; Zhang et al., 2019; Dong et al., 2021; Yu et al., 2022a). These methods often use a rectangle representation *g* = (*x, y, h, w*, θ), where (*x, y*) is the center pixel location of a grasp candidate, (*h, w*) are height and width of the gripper, and θ is the rotation of the gripper. This representation is widely used in end-to-end deep networks. Some other studies (Wang et al., 2019, 2022) also used a score map with the same size as the image to represent the quality of grasp configurations at each pixel.

A number of existing grasp detection methods are inspired by object detection (Zhou et al., 2018; Zhang et al., 2019; Park et al., 2020). These two problems share a similar output, which consists of a regression of a rectangle (a grasp configuration for grasp detection or a bounding box for object detection) and a classification score (quality of the grasp or confidence in the predicted category). Thus, one straightforward way for designing a grasp detection model is to modify it from an object detector. For example, Zhang et al. (2019) propose an ROI-based grasp detection method which is a modification from Faster R-CNN. They use the region proposal network (RPN) to generate graspable proposals and an ROI-pooling layer to extract features for each proposal. Then grasp configurations and corresponding successful rates are estimated with the local features. However, grasp detection differs from object detection in the additional prediction of orientation. To predict the orientation of the gripper, serval existing methods (Chu et al., 2018; Dong et al., 2021; Yu et al., 2022a) convert this regression problem into a classification problem by discretizing continuous angles into angle anchors. This makes orientation prediction much more convenient but will also cause a loss of accuracy. To overcome this short back, other studies (Park et al., 2020) use classification and regression processes to predict the final angles. Another kind of grasp detection method (Yu et al., 2022b) makes dense predictions at each pixel and outputs a set of heatmaps representing the grasp configurations and quality.

To generate more reasoning and human-like grasp candidates, we investigate the problem of simultaneously detecting objects and grasp configurations from an RGB-D image and propose a novel neural learning approach, namely SOGD, for this task. Our SOGD model takes an RGB-D image as input and outputs a set of tuples (*x*_*o*_, *y*_*o*_, *w*_*o*_, *h*_*o*_, *cls*_*o*_, *x*_*g*_, *y*_*g*_, *w*_*g*_, *h*_*g*_, θ_*g*_, *s*_*g*_), representing the joint prediction of the object detection result and the grasp detection result. To this end, features extracted by the top stages of a backbone and feature pyramid network (FPN) are used for both detection tasks. Two separate detection branches are designed to detect objects and grasp them, respectively. The correspondences between object proposals and grasp candidates are modeled by an alignment module. In addition, we present a depth-based method to filter out backgrounds in a cluttered scene. This would enable our detectors to focus on features from the target objects other than the texture from the environment.

Our main contributions are summarized as follows:

(1) We propose a novel neural learning approach to detect target objects and their best grasp configurations in cluttered environments simultaneously.
(2) An alignment module is designed to estimate the correlations between the separately detected objects and grasp configurations. This module enables our model to predict more reasonable grasp configurations for each detected object.
(3) A 3D-plane-based pre-processing is presented to filter out cluttered backgrounds from the RGB-D image.
(4) A series of experiments are conducted on two publicly available datasets (the Cornell Grasp Dataset and the Jacquard Dataset). Our method achieves +0.7 to +1.4% improvement in average accuracy compared with the existing RGB-D-based grasp detection methods.

## 2. Related studies

Grasp detection methods can be divided into traditional methods and learning-based methods. The traditional methods are mainly divided into the template matching method and the feature point matching method. The template-based pose estimation algorithm (Georgakis et al., 2019) needs to build the template of the object in advance, which can be strongly applicable to objects with regular shapes and has a good effect on targets without texture. However, when the object is blocked and the light is insufficient, the matching will be too low, leading to the failure of prediction. The pose estimation method based on feature points can extract effective feature points from images and match them with standard images. Since descriptors can describe visual features stably and robustly, this method is not susceptible to illumination. However, this method only uses the information of feature points in the image, so the utilization rate of information is very low. If there are not many feature points in the image, this method will have a high probability of deviation from the predicted capture rectangle.

Motivated by the superior performance of deep learning technology (Chhabra et al., 2022; Motwani et al., 2022; Shailendra et al., 2022; Singh et al., 2022), it has been applied in grasping detection to improve the accuracy of grasping in recent years. In order to improve the generalization of 3D models, some grab detection methods based on 3D reconstruction are proposed. According to Yang et al. (2021), this method uses 3D reconstruction to optimize the candidate grasping objects generated by the grasping suggestion network and improves the grasping accuracy of unknown objects. According to Jiang et al. (2021), this method uses implicit neural representation and studies synergies between affordance and geometry to improve the accuracy of grasping detection. Sundermeyer et al. (2021) used a 3D point cloud to predict the 3D points of grasping contact and reduce the dimension from 6-Dof to 4-Dof to make the learning process more convenient. However, the method based on 3D reconstruction needs a certain amount of time to build the 3D model, and the real-time capturing will be affected to some extent.

Since deep learning has shown excellent results in detection and segmentation tasks, image-based capture detection methods have become increasingly popular. But different from object detection, grasp detection needs to predict the angle of the gripper. Therefore, some of the methods (Chu et al., 2018; Dong et al., 2021; Yu et al., 2022a) discretized continuous angles into angle anchors and transformed the regression problem into a classification problem. However, these methods may cause a lack of accuracy. To solve this problem, Park et al. (2020) predicted the final angle using classification and regression processes. The method provided by Yu et al. (2022b) intensively predicts that each pixel represents the heat map of the capture configuration and quality. Zhang et al. (2018) divided the grasping problem into two separate tasks (object detection and grab detection) and then integrated them as the final solution. Yu et al. (2022b) proposed a module that extracts feature mappings from bidirectional feature pyramid networks, object detection, and grab detection, and outputs the optimal grab position and appropriate operational relations. Park et al. (2020) predicted the boundary box, the category of objects, and the direction of the grab rectangle and grab configuration using a global feature map. Ainetter and Fraundorfer (2021) designed an end-to-end CNN-based network architecture and designed a refinement module to improve the accuracy of prediction.

Most deep-learning-based methods directly output grasp candidates without recognizing the target object. They cannot answer the question that what is the best grasp configuration for every single object in a cluttered scene. Unfortunately, this is a common case an unmanned system needs to deal with. To generate a more reasoning prediction, Zhang et al. (2018) designed a recognize-and-then-grasp approach, which divides the problem into two separate tasks (object detection and grasp detection) and then integrates them as the final solution. Another way is to perform grasp detection together with object detection or segmentation tasks (Park et al., 2020; Ainetter and Fraundorfer, 2021; Yu et al., 2022a). For example, Park et al. (2020) generated a global feature map to predict the bounding box, the category of an object, the grasp rectangle, and the orientation of a grasp configuration. Then, non-maximum suppression is applied to both bounding boxes and grasp rectangles to filter out unnecessary predictions. The relationship between the bounding boxes and the grasp rectangles is built *via* computing the Intersection over Union (IoU) of the two areas. If the IoU is greater than a certain threshold, the grasp will be assigned to the detected object. However, choosing an appropriate threshold is not easy. When the graspable area is much smaller than the entire object, the IoU between the grasp rectangle and the bounding box of the object will be too small. As a result, such a strategy cannot avoid filtering out possible solutions.

## 3. Materials and methods

### 3.1. Problem formulation and reparameterization

In the process of object grabbing, humans usually first identify the object to be grabbed in the scene and then select an appropriate grabbing position for the target object. Whether a grasping pose is appropriate is directly related to the target object to be grasped. Motivated by this fact, this study investigates the problem of robotic manipulation by detecting the target object in the scene and its grasp position simultaneously.

Given an RGB-D image, the goal of the simultaneous object and grasp detection is to identify every single object in the scene and find out a grasp configuration for it. To this end, we formulate the representation of the simultaneous object and grasp detection **det** as:


det = (od, gd) 

$$od\;=\;\left(x_{o},\;y_{o},\;w_{o},\;h_{o},\;cls_{o}\right)$$

$$gd\;=\;\left(x_{g},\;y_{g},\;w_{g},\;h_{g},\;{\text{θ}}_{g},\;s_{g}\right)s$$

**Figure 1:**
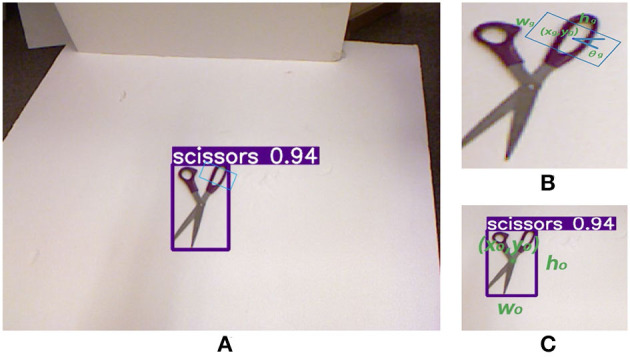
An example of our simultaneous object and grasp detection representation. **(A)** 11D object and grasp detection representation. **(B)** 5D object detection representation with location (*x*_*o*_, *y*_*o*_), width *w*_*o*_, height *h*_*o*_, and the category of the object *cls*_*o*_. **(C)** 6D grasp detection representation with location (*x*_*g*_, *y*_*g*_), gripper width *w*_*g*_, plate size *h*_*g*_, orientation θ_*g*_, and its success rate *s*_*g*_.

The representation consists of two parts: object detection and grasp detection. Figure 1 shows an example of this representation. For the object detection part, we use (*x*_*o*_, *y*_*o*_, *w*_*o*_, *h*_*o*_) to represent the location of a bounding box and *cls*_*o*_ to represent the category of the object inside of it. For the grasp detection part, we adopt the famous 5-dimensional rectangular representation (Lenz et al., 2013), which consists of the location and orientation (*x*_*g*_, *y*_*g*_, *w*_*g*_, *h*_*g*_, θ_*g*_) of the gripper for a grasp configuration. In addition, we add *s*_*g*_, a value between 0 and 1, to represent the score of a grasp. The higher *s*_*g*_ is, the greater chance of the grasp being a success.

Similar to Park et al. (2020), we formulate the estimation of θ_*g*_ as a classification + regression problem instead of a single regression problem. According to the symmetry of the gripper, the range of θ_*g*_ is [0, π]. We convert this range into several bins $\left\{0,\frac{\pi }{k_{a}},\frac{2\pi }{k_{a}},\;...,\left(k_{a}-1\right)\frac{\pi }{k_{a}}\right\}$ to be angle anchors, with *k*_*a*_ is the number of bins. The classification problem is to predict a one-vs.-all vector to determine which bins θ_*g*_ belongs to. The regression problem is to estimate the angle offset to the anchors.

Inspired by Redmon and Farhadi (2018) and Ge et al. (2021), we reparametrize the regression problem of (*x*_*j*_, *y*_*j*_, *w*_*j*_, *h*_*j*_, θ_*j*_) as estimation of $\left(t_{j}^{x},t_{j}^{y},t_{j}^{w},t_{j}^{h},t_{j}^{\theta }\right)$ to the location of the grid cell $\left(a_{j}^{x},a_{j}^{y}\right)$, bounding box prior width and height $\left(a_{j}^{w},a_{j}^{h}\right)$, and orientation angle bin $a_{j}^{\theta }$. The relationship between (*x*_*j*_, *y*_*j*_, *w*_*j*_, *h*_*j*_, θ_*j*_) and $\left(t_{j}^{x},t_{j}^{y},t_{j}^{w},t_{j}^{h},t_{j}^{\theta }\right)$ is defined as follows.


$$\begin{array}{l} x_{j}\;=\;\sigma \left(t_{j}^{x}\;\right)+\;a_{j}^{x}\; \end{array}$$

$$\begin{array}{l} y_{j}\;=\;\sigma \left(t_{j}^{y}\;\right)+\;a_{j}^{y}\; \end{array}$$

$$\begin{array}{l} w_{j}\;=\;a_{j}^{w}\;\times \;e^{t_{j}^{w}}\; \end{array}$$

$$\begin{array}{l} h_{j}\;=\;a_{j}^{h}\;\times \;e^{t_{j}^{h}}\; \end{array}$$

$$\begin{array}{l} \theta_{j}\;=\;\sigma \left(t_{j}^{\theta }\right)\times \left(\frac{\pi }{k_{a}}\right)\;+\;a_{j}^{\theta }\; \end{array}$$

This reparameterization is applied to both the object bounding box regression and the grasp rectangle regression.

### 3.2. Overview of the SOGD model

**Figure 2:**
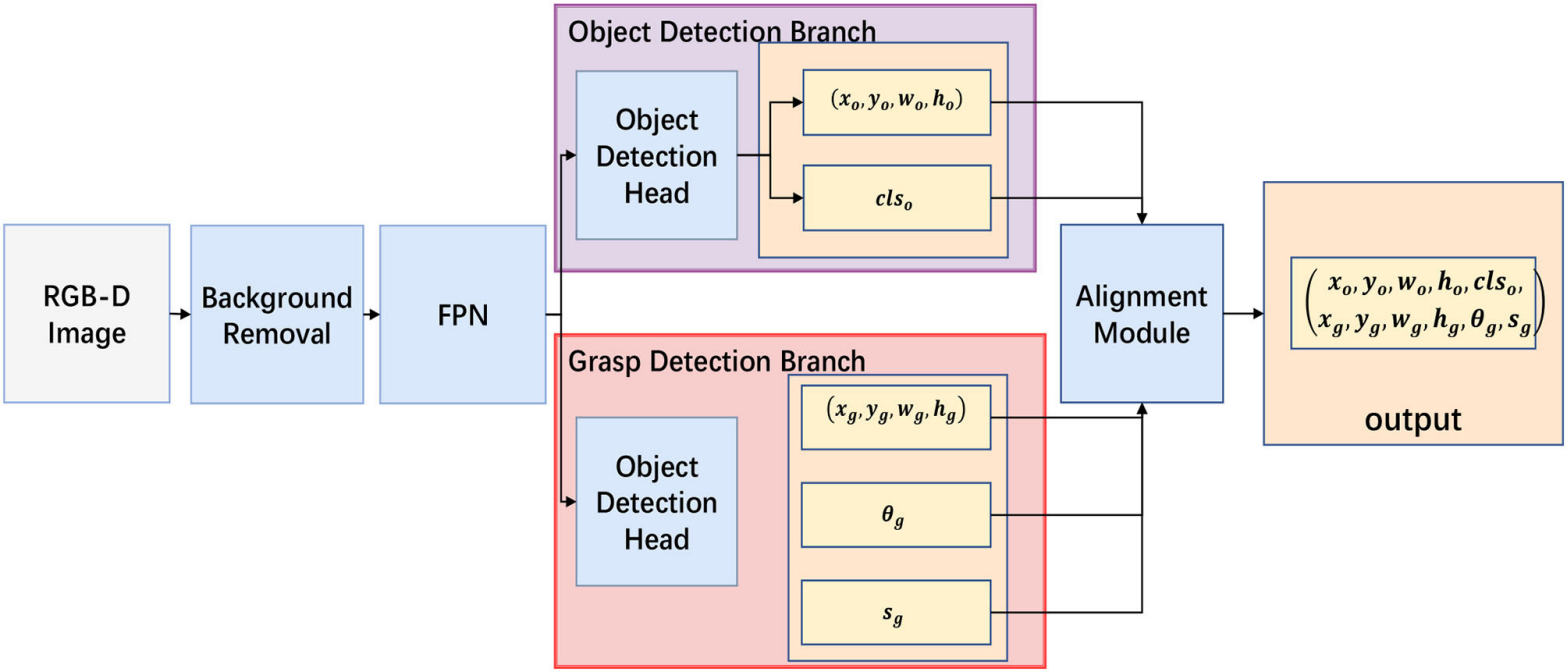
Outline of our Simultaneous Object and Grasp Detection (SOGD) model. SOGD takes an RGB-D image as input and outputs a series of recognized objects together with the most appropriate grasp for every single object. It consists of five parts: a pre-processing module to remove background from the cluttered scene, a backbone (e.g., Darknet or ResNet) and FPN for hierarchical feature extraction, two separate branches for object detection and grasp detection, and an alignment module to assign a most appropriate grasp for each detected object.

The architecture of our SOGD model is shown in Figure 2. It takes an RGB-D image as input, and outputs the detected object's bounding $\left(t_{o}^{x},t_{o}^{y},t_{o}^{w},t_{o}^{h}\right)$ and category *cls* together with the corresponding grasp position $\left(t_{g}^{x},t_{g}^{y},t_{g}^{w},t_{g}^{h}\right)$, orientation $t_{g}^{\theta }$, and success rate *s*_*g*_. Our model consists of five parts: a pre-processing for background removal, a backbone and a feature pyramid network (FPN) for image feature extraction, an object detection head, a grasp detection head, and an alignment module for candidate objects and grasp configurations.

Motivated by Dong et al. (2021), we design a pre-processing module to remove the background from the cluttered scene. According to Dong et al. (2021), backgrounds are recognized by an encoder–decoder network to segment the original image. However, our module utilizes the priors of the scene that objects to be grabbed are laid on a 3D plane, such as the surface of a desk. According to this fact, we categorize pixels on and under the 3D plane as background and filter them out. We argue that this background removal strategy is more reasonable and robust than the U-net-based method (Dong et al., 2021). Details about the 3D-plane-based background removal are discussed later.

For multi-scale feature extraction, various deep models [e.g., Darknet (Wood, 2009) or ResNet (He et al., 2016)] can be utilized. In this study, ResNet-50 is used as the backbone to release the computational burden of deep models during feature extraction and facilitate real-time performance. The very last feature map learned by different stages (e.g., *conv1, conv2*, and *conv3*) is used as multi-scale features. We denote these feature maps as {*C*_1_, *C*_2_, *C*_3_, *C*_4_, *C*_5_}. The stride steps of these feature maps are {2, 4, 8, 16, 32} with respect to the original image. Only {*C*_3_, *C*_4_, *C*_5_} are used in FPN for feature fusion and the fused feature maps are denoted as {*P*_3_, *P*_4_, *P*_5_}. Our feature extraction and fusion module can be formulated as follows:


$$\begin{array}{l} image\text{\_}f\;=\;BackgroundRemoval\left(image\text{\_}rgbd\right)\; \end{array}$$

$$\begin{array}{l} F_{backbone}\;=\;\left\{C_{3},\;C_{4},\;C_{5}\right\}\;=\;ResNet\left(image\text{\_}f\right)\; \end{array}$$

$$\begin{array}{l} F_{FPN}\;=\;\left\{{\text{P}}_{3},\;{\text{P}}_{4},\;{\text{P}}_{5}\right\}\;=\;FPN\left(F_{backbone}\right)\; \end{array}$$

Inspired by the fact that grasp rectangles are often much small than the object's boundaries, we use different prior rectangle sizes for object detection and grasp detection. The object detection head and the grasp detection head share a similar structure with the detection head (Redmon and Farhadi, 2018). The output tensor for object detection is in the shape of *N*×*N*×*k*_*o*_×(4 + 1+*C*_*o*_), where *N*×*N* is the size of the feature map, *k*_*o*_ is the number of predicted bounding boxes, 4 stands for the number of parameters of a bounding box, 1 stands for the channel of confidence, and *C*_*o*_ is the number of object categories. Similarly, the output tensor for grasp detection is in the shape of *N*×*N*×*k*_*g*_×(5+*C*_*a*_+1), where 5 stands for the location, width, height, and orientation of a grasp rectangle, *C*_*a*_ is the number of angle bins, and 1 stand for the successful rate prediction.

### 3.3. Background removal

**Figure 3:**
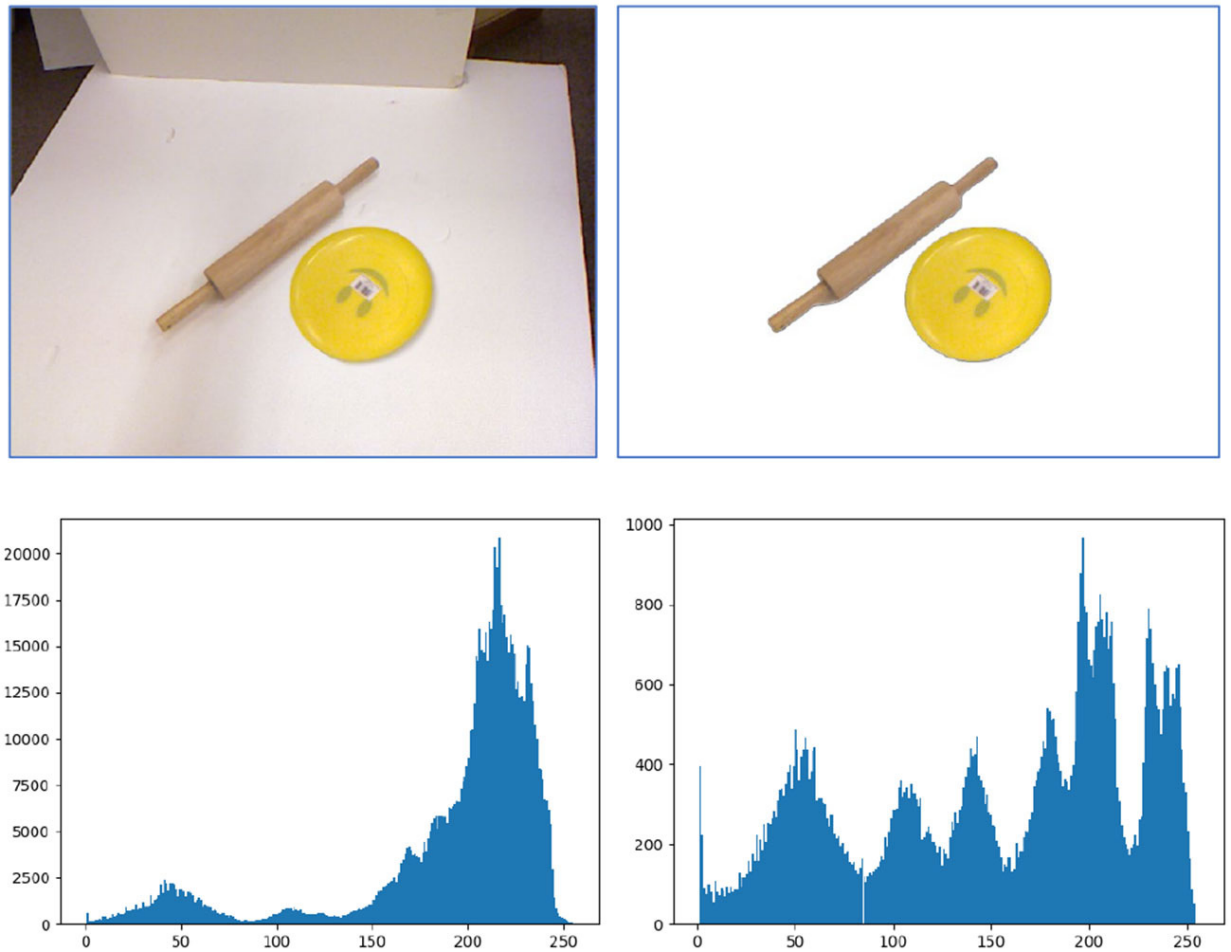
Illustration of the unnecessary efforts spent on the cluttered background during feature extraction. **Top** lines are original images and foreground images. **Bottom** lines are the corresponding histograms of the top images. From the histograms, it can be seen that the additional information which is useless to target detection will significantly increase when a cluttered background is encountered in the image.

Robotic manipulation often encounters a cluttered environment. The captured RGB-D images include both the target objects to be grasped and the background surroundings. To achieve an accurate object and grasp detection, we should focus on the pixels belonging to the targets. The additional observation on backgrounds may distract our attention from the targets. Figure 3 presents a quantitative analysis of this additional information. When the background is removed, the detection model only needs to learn features from the target objects and all learned features are valuable for final manipulation. However, if the RGB-D image encounters a cluttered background, the model will have to learn features from both the targets and the background, and distinguish which feature contributes to the downstream tasks. This will increase the burden of the model for feature extraction and also increase the number of parameters to learn task-specific features. According to Dong et al. (2021), these additional cluttered background pixels will even lead to a false grasp detection.

To eliminate the cluttered background and let the model focus on the targets, Dong et al. (2021) adopt an encoder–decoder-based U-net model to segment the input image into foreground and background. It is indeed a potential way to filter out the background in an image. But the U-net model needs to be trained on a large dataset and its generalizability to new observations is limited. Instead of recognizing the background in the image domain, we present a background removal method in 3D space. We assume that objects to be grasped are laid on a 3D plane (such as the surface of a desk), which is the common case in robotic manipulation. Under this assumption, pixels that are up to the 3D plane are defined as the foreground, while pixels in or under the 3D plane are defined as the background. This could separate the targets from the cluttered background in most cases. In the top-left image in Figure 3, the white vertical surface will also be considered as the foreground using the aforementioned approach. But this mis-segmentation will not affect the detection of the targets since the vertical surface is disconnected from the targets.

For the 3D plane estimation, we use a model-based method to fit the unknown parameters. In 3D spaces, a plane is defined as *aX*+*bY*+*cZ*+*d* = 0, with (*a, b, c, d*) as the plane parameters. Given three points, we can fit a plane for it. Since pixels belonging to the 3D plane are dominant in the image, we adopt a RANSAC-based method to fit the parameters of the largest 3D plane in the image. The method achieves its goal by iteratively selecting a random subset of the original 3D points. The selected subset is assumed to be inliers and the plane parameters are fitted with respect to these inlier points. Then all other points are tested against the fitted model. If a point fits well to the estimated model, it will be considered as inliers to the model. The fitted 3D plane is reasonably good if sufficiently many points are classified as inliers. In this study, 3D coordinates are computed from the depth image with a fixed camera intrinsic parameter when 3D points are not presented in the dataset. We also applied voxelization to speed up the process and generate a finer plane.

### 3.4. Separate object and grasp detection branches

**Figure 4:**
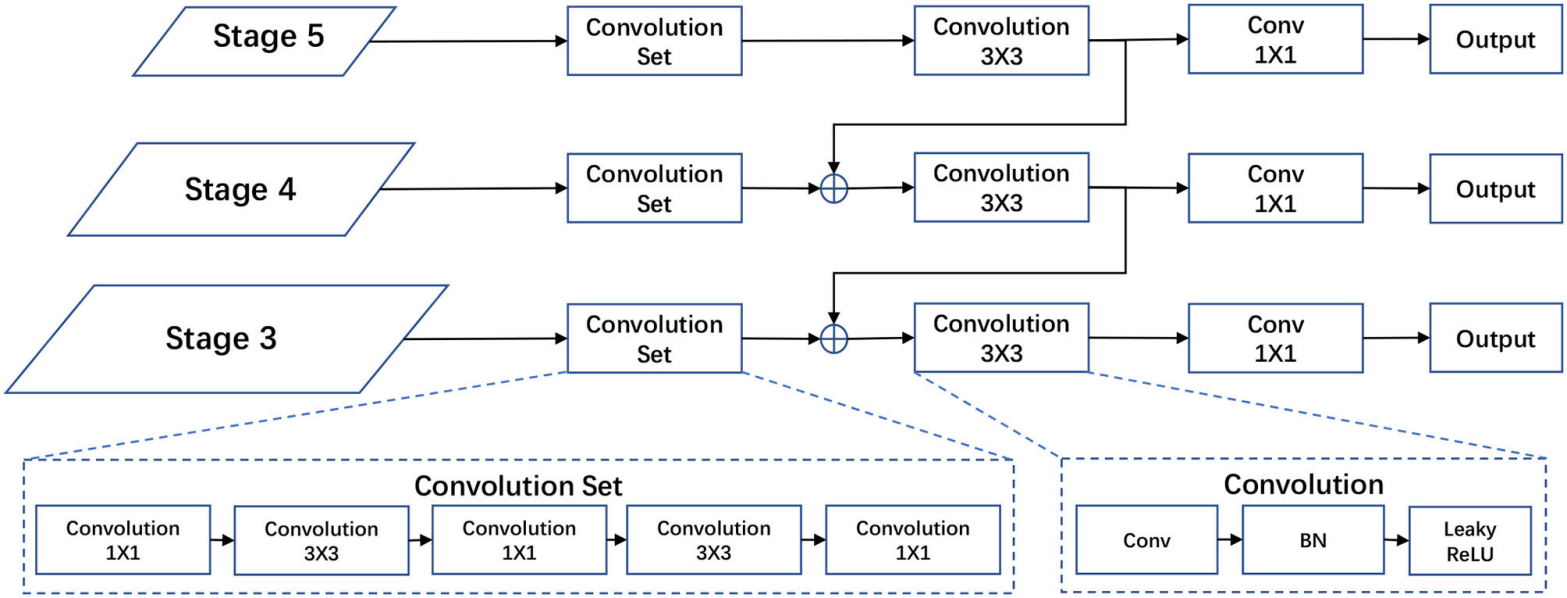
Structure of the object/grasp detection head. Our object detection head and grasp detection head share a similar structure except for the output channels. Both heads take {*P*_3_, *P*_4_, *P*_5_} from the FPN as inputs. The output of object detection includes the bounding box and object categories, while the output of grasp detection has more channels for orientation and grasp score.

Object detection and grasp detection are both detection problems. These two tasks share a similar output in the regression of location, width and height of a rectangle (as the bounding box for object detection and the grasp rectangle for grasp detection), and a confidence score (as the classification for object detection and the grasp quality for grasp detection). According to observation, we design two separate detection branches for these two tasks, but the branches share a similar architecture as shown in Figure 4.

The structure of our two detection branches is motivated by modern object detectors (Redmon and Farhadi, 2018). The detection head takes multi-stage outputs from FPN as inputs to detect objects or grasp configurations at different scales. In fact, a gripper only needs to grab a small part of an object to take it up, instead of grabbing the whole of it. As a result, the detected rectangle for grasp is often smaller than the bounding box of the object. Thus, we use the same inputs as modern detectors for object detection and grasp detection, but a relatively small-scale prior size for grasp detection. This enables our model to use a same-level semantic feature for both tasks.

Our detection head consists of a convolution set, a 3 × 3 convolution block, and a 1 × 1 convolution layer for prediction. The multi-stage outputs of FPN are treated as fused features which include both texture and semantic information extracted from the input image. The convolution set is designed to learn a task-correlated feature representation from the texture and semantic information. Then the 3 × 3 convolution block fuses task-corelated features at the top and current scales. The 1 × 1 convolution layer is used to match the number of channels to the final predictions. The number of channels of outputs for object detection is *k*_*o*_×(4 + 1+*C*_*o*_), where *k*_*o*_ is the number of predicted bounding boxes for each grid cell; 4 stands for $\left(t_{o}^{x},t_{o}^{y},t_{o}^{w},t_{o}^{h}\right)$, parameters of a bounding box; 1 stands for the confidence of the prediction; and *C*_*o*_ is the number of object categories. Similarly, the number of channels of output for grasp detection is *k*_*g*_×(5+*C*_*a*_+1), where *k*_*g*_ is the number of predicted grasp rectangles; 5 stands for $\left(t_{g}^{x},t_{g}^{y},t_{g}^{w},t_{g}^{h},t_{g}^{\theta }\;\right)$, parameters of a grasp rectangle; 1 stands for the score of the grasp configuration; and *C*_*a*_ is the number of angle bins. The mathematical computation of our detection head is as follows:


$$F_{task}\;=\;\left\{T_{3},T_{4},T_{5}\right\}\;=\;ConvolutionSet\left(F_{FPN}\right)\;$$

$$F_{task\_fusion}\;=\;\left\{TF_{3},TF_{4},TF_{5}\right\}\;$$

$$TF_{i}\;=\;Convolution\left(TF_{i+1}\;+\;T_{i}\right)\;$$

$$Pro\;=\;Conv_{1\times 1}\left(F_{task\_fusion}\right)\;$$

### 3.5. Alignment between objects and grasp configurations

The two detection branches make predictions for objects and grasp configurations separately. To model the correspondence between detected objects and grasp configurations, we design an alignment module. Given an object prediction $Pro_{o}\in R^{N\times N\times k_{o}}$ and a grasp prediction $Pro_{g}\in R^{N\times N\times k_{g}}$, the correspondences between all possible pairs are defined as ${\text{P}r\text{o}}_{\text{c}or\text{r}}\in {\text{R}}^{\left(N\times N\times k_{o}\right)\times \left(N\times N\times k_{g}\right)}$. Objects and grasp configurations are detected at different scales. Generating correspondences across multi scales would significantly increase the computational complexity. Thus, we only consider possible object-grasp pairs within the same scale.

Our alignment module takes the task-correlated features from object detection head ${\text{T}\text{F}}_{\text{o}}\in {\text{R}}^{N\times N\times c_{o}}$ and grasp detection head ${\text{T}\text{F}}_{g}\in {\text{R}}^{N\times N\times c_{g}}$ as input. Then two 1 × 1 convolution layers are applied to the features separately, resulting in the outputs of ${\overset{´}{\text{F}}}_{\text{o}}\in {\text{R}}^{N\times N\times k_{o}\times f}$ and ${\overset{´}{\text{F}}}_{g}\in {\text{R}}^{N\times N\times k_{g}\times f}$. The two feature maps are reshaped into a 2D matrix and transpose matrix multiplication is applied to generate the output ${\text{F}}_{\text{c}or\text{r}}\in {\text{R}}^{\left(N\times N\times k_{o}\right)\times \left(N\times N\times k_{g}\right)}$. Finally, we use a sigmoid activation function to model the joint possibility of the detection pairs. The mathematical computation of our alignment module can be formulated as follows:


$$F_{o}\;=\;Conv_{1\times 1}\left(TF_{o}\right)\;$$

$$F_{\text{g}}\;=\;Conv_{1\times 1}\left(TF_{g}\right)\;$$

$$F_{corr}\;=\;reshape(F_{o})•{\left(reshape(F_{g})\right)}^{T}\;$$

$$Pro_{corr}\;=\;sigmoid(F_{corr})\;$$

Though our two detection heads make predictions separately, our model is forced to learn a better **Pro**_*corr*_ to model the correlation between the predictions. At inference, we use two additional parameters *k*_*o*_ and *k*_*c*_ to control the number of final predictions. First, top *k*_*o*_ object predictions are selected from **Pro**_*o*_, then top *k*_*c*_ correlated grasp predictions are selected and assigned to the detected objects. If the quality of a grasp prediction is smaller than a threshold, the grasp prediction will be filtered out from the alignment results. In this way, our model can be easily extended to multi-object detection and multi-grasp detection cases.

### 3.6. Loss function

The loss of our SOGD model consists of three parts: object detection loss *L*_*o*_, grasp detection loss *L*_*g*_, and alignment loss *L*_*corr*_. The loss of object detection is defined as:


$$\begin{array}{l} L_{o}\;=\;L_{o}^{reg}\;+\;\alpha \;\times \;L_{o}^{cls}\; \end{array}$$

$$\begin{array}{l} L_{o}^{reg}\;=\;\frac{1}{N_{o}^{reg}}\underset{i}{\sum }{smooth}_{L1}\left(t_{o}^{i},{\hat{t}}_{o}^{i}\right)\; \end{array}$$

$$\begin{array}{l} L_{o}^{cls}\;=\;\frac{1}{N_{o}^{cls}}\underset{i}{\sum }{Loss}_{focal}\left({cls}_{i},{\hat{cls}}_{i}\right)\; \end{array}$$

where $t_{o}^{i}$ and ${\hat{t}}_{o}^{i}$ are ground truth and predictions for a bounding box, respectively. We use the smooth L1 loss for regression and focal loss (Lin et al., 2017) for classification. $N_{o}^{reg}$ and $N_{o}^{cls}$ are the normalizers. α is the weight of classification loss.

Similarly, the loss of grasp detection is defined as:


$$\begin{array}{l} L_{g}\;=\;L_{g}^{reg}\;+\;\beta \;\times \;L_{g}^{angle}\;+\;\gamma \;\times \;L_{g}^{score}\; \end{array}$$

$$\begin{array}{l} L_{g}^{reg}\;=\;\frac{1}{N_{g}^{reg}}\underset{i}{\sum }{smooth}_{L1}\left(t_{g}^{i},{\hat{t}}_{g}^{i}\right)\; \end{array}$$

$$\begin{array}{l} L_{g}^{angle}\;=\;-\underset{i}{\sum }\left[a_{i}\log\left({\hat{a}}_{i}\right)\;+\;\left(1-a_{i}\right)\log\left(1-{\hat{a}}_{i}\right)\right]\; \end{array}$$

$$\begin{array}{l} L_{g}^{score}\;=\;-\underset{i}{\sum }\left[s_{i}\log\left({\hat{s}}_{i}\right)\;+\;\left(1-s_{i}\right)\log\left(1-{\hat{s}}_{i}\right)\right]\; \end{array}$$

where $t_{g}^{i}$ and ${\hat{t}}_{g}^{i}$ are ground truth and predictions for a grasp rectangle, respectively. β and γ are hyperparameters.

The loss of object and grasp alignment is defined as:


$$\begin{array}{l} L_{corr}\;=\;-\delta \times \underset{i}{\sum }\left[p_{i}\log\left({\hat{p}}_{i}\right)\;+\;\left(1-p_{i}\right)\log\left(1-{\hat{p}}_{i}\right)\right]\; \end{array}$$

where *p*_*i*_ and ${\hat{p}}_{i}$ are ground truth and predictions of a candidate pair for object and grasp, respectively. δ controls the weights of alignment loss to the total loss.

The total loss function is the summation of the three losses:


$$\begin{array}{l} L\;=\;L_{o}\;+\;L_{g}\;+\;L_{corr} \end{array}$$

## 4. Results

To evaluate the performance of our proposed SOGD model against previous methods, we test it on two publicly available datasets: the Cornell Grasp Dataset (Lenz et al., 2013) and the Jacquard Dataset (Depierre et al., 2018). Our model is designed for the task of grasp detection, but it also outputs predictions for object detection. Thus, the metrics used in our experiments consist of two parts. For the grasp detection task, we use the popular Jaccard Index and angle difference as metrics, consistent with previous methods (Jiang et al., 2011; Chu et al., 2018; Kumra et al., 2020; Yu et al., 2022b). A predicted grasp configuration is considered as correct if and only if the following two conditions are satisfied.

(1) Jaccard Index of the predicted grasp rectangle and the ground truth is >0.25. Assuming that ${\hat{b}}_{g}$ is the predicted grasp rectangle and *b*_*g*_ is the ground truth, Jaccard Index is defined as:


$$\begin{array}{l} Jaccard\;Index\;=\;\frac{\left|{\hat{b}}_{g}\cap b_{g}\right|}{\left|{\hat{b}}_{g}\cup b_{g}\right|} \end{array}$$

(2) the difference between the predicted orientation angle and the ground truth is < 30°.

For object detection tasks, we use accuracy instead of the commonly used mAP as metrics. In this research, object detection is an additional output only to achieve the final goal of predicting the most possible grasp for each individual object in a cluttered scene. Our method only needs to know where the object is, and what kind of object it is does not matter too much. So, we consider a prediction as correct if the intersection over union of the predicted bounding box and ground truth is >0.5.

### 4.1. Grasp detection results on cornell grasp dataset

There are 878 images together with the corresponding depth image and 3D point clouds in the Cornell Grasp Dataset (Lenz et al., 2013). The resolution of the images is 640 × 480. Each image contains a single graspable object at different positions and orientations. The dataset is manually annotated with many positive and negative grasp rectangles. Following previous research (Zhang et al., 2019; Dong et al., 2021), we use a five-fold cross-validation strategy to evaluate the performance of our method and report the average detection accuracy in this section. The reported results include both image-wise (IW) and object-wise (OW) detection accuracy. In image-wise experiments, all images are randomly divided into a train set and a test set. The object in the test set may have been learned during training but at different poses and views. This is mainly to test the generalization ability of our method when objects are captured from multiple points of view. In object-wise experiments, images are divided according to the object categories. Objects in the test set have never been seen during training. This is to test the generalization ability of our method when it faces a new kind of object.

The evaluation of grasp detection accuracy and efficiency are summarized in Table 1. Our method achieves 98.9 and 98.3% accuracy on image-wise and object-wise detection tasks, respectively. Compared with the state-of-the-art RGB-D-based method (Yu et al., 2022b), our SOGD shows an improvement of +0.7 and +1.2% in accuracy. The efficiency of our method is relatively low than SE-ResUNet (Yu et al., 2022b). In SE-ResUNet, a squeeze-and-excitation residual network is designed to predict the width and orientation of the grasp rectangle and the quality of the grasp outputs. It does not involve the detection of the target object. As a result, the complexity of SE-ResUNet is smaller than ours and their detection ability is not strong as ours. Similar results are observed when our SOGD is compared with Kumra et al. (2020). Compared with the state-of-the-art RGB-based method (Yu et al., 2022a) and RG-D-based method (Park et al., 2020), our SOGD shows a similar performance in the image-wise detection task, but a superior performance in the object-wise detection task. This is mainly because our SOGD not only learns possible grasp configurations but also the correspondences between the target object and grasp candidates. In this way, it has the ability to figure out what is the best grasp configuration for a specific kind of object. Therefore, when facing new objects, it can benefit from learned knowledge of the relationship between grasp configurations and objects.

**Table 1:** Grasp detection results on the Cornell Grasp Dataset.

**Methods**	**IW/%**	**OW/%**	**FPS**	**Input**
Chu et al. (2018)	94.4	95.5	8.3	RGB
Wang et al. (2021)	96.1	95.5	-	RGB
Asif et al. (2019)	96.7	-	-	RGB
Yu et al. (2022a)	**98.9**	**97.8**	**50.0**	RGB
Dong et al. (2021)	96.4	96.5	9.4	RG-D
Song et al. (2020)	95.6	97.1		RG-D
Zhang et al. (2019)	92.3	91.7	25.2	RG-D
Park et al. (2020)	**98.6**	**97.8**	**62.5**	RG-D
Jiang et al. (2011)	60.5	58.3	0.2	RGB-D
Lenz et al. (2013)	73.9	75.6	0.7	RGB-D
Chu et al. (2018)	96.0	96.1	8.3	RGB-D
Kumra et al. (2020)	97.7	96.6	**50.0**	RGB-D
Yu et al. (2022b)	98.2	97.1	40.0	RGB-D
SOGD (ours)	**98.9**	**98.3**	9.6	RGB-D

The best performance of methods within a same kind of input is shown as bold.

**Figure 5:**
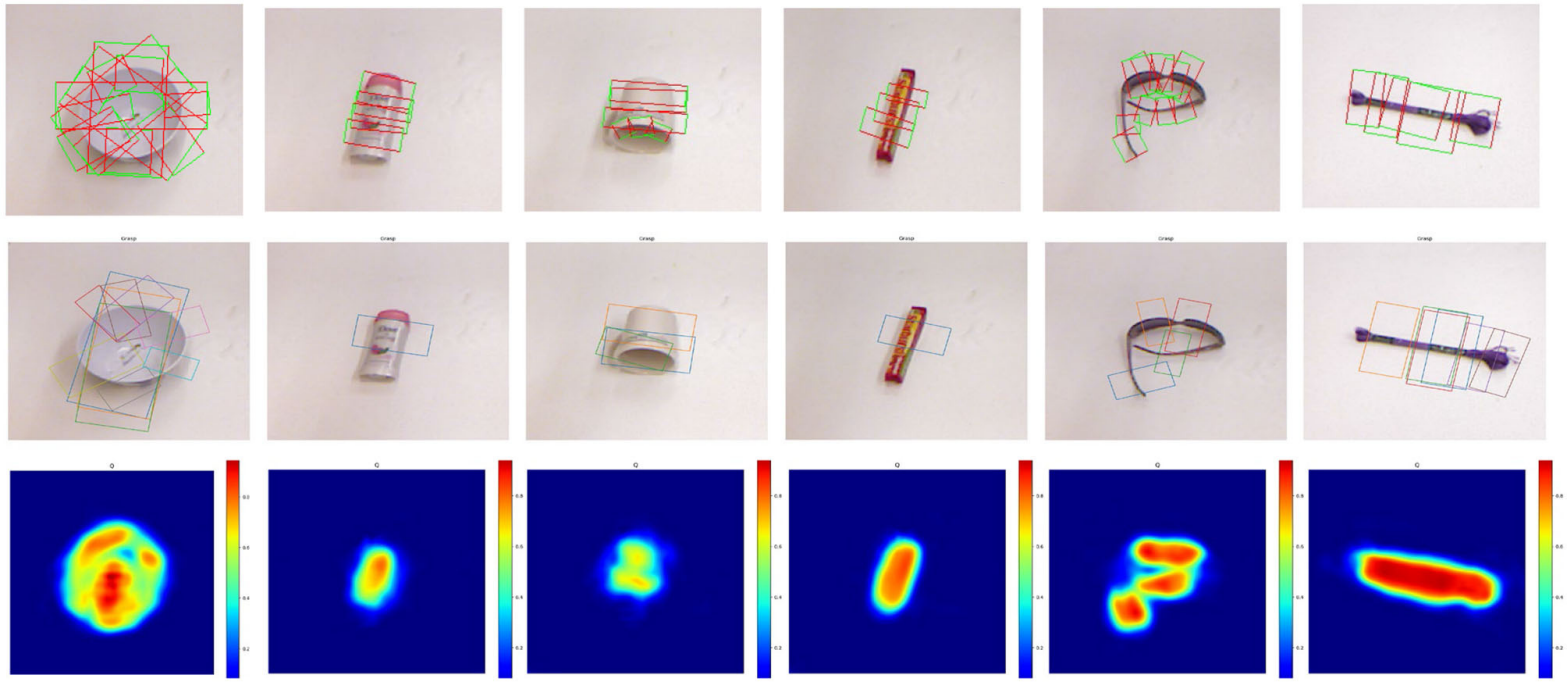
Typical grasp detection results of our SOGD on the Cornell Grasp Dataset. **Top** line is ground truth annotation; **middle** line is the prediction of our SOGD; and **bottom** line is the estimated grasp quality of the corresponding detection.

Visualization of typical grasp detection results is shown in Figure 5. The three lines in the figure are ground truth annotations, predicted grasp configurations of our SOGD, and the corresponding grasp quality predicted by our SOGD. For each detected object, our model outputs the best grasp rectangle for it. In this experiment, the quality is not the direct output from the grasp detection branch in our SOGD model. Our grasp detection branch outputs a score map for each grasp candidate that can be treated as the quality of the grasp, as mentioned in a previous study (Yu et al., 2022a,b). But our model includes an alignment module to learn the correspondences between predicted objects and grasp configurations. The quality is the product of the score map and the correspondences. It represents the success rate of a grasp if there is a detected target object. From the figure, it can be seen that our SOGD has a good ability in detecting grasp rectangles and the output quality map is able to provide a clear reason for the grasp decision.

### 4.2. Grasp detection results on Jacquard Dataset

The Jacquard Dataset (Depierre et al., 2018) is collected from a simulator with CAD models of the ShapeNet dataset. There are 54k images with 11k different kinds of objects in the dataset. A large number of samples facilitate our model training. However, we still use some data augmentation strategies (like random rotation) to increase the robustness of the learned model. The resolution of images in this dataset is 1,024 × 1,024. We down-sample the original image to a size of 512 × 512 for both training and testing. Unlike results on the Cornell Dataset, we report the overall accuracy of the grasp detection results on the Jacquard Dataset.

Table 2 reports the performance of our SOGD against the state-of-the-art methods. Our SOGD shows a 99% accuracy on the Jacquard Dataset, which is higher than both RGB-based and RG-D-based state-of-the-art methods. Compared with MASK-GD (Dong et al., 2021), our method achieves a +1.4% performance boost. MASK-GD also involves pre-processing for background removal. Background removal is treated as an image segmentation problem and a deep network is trained for it in MASK-GD. This strategy has the potential to recognize the foreground targets from the cluttered scenes, and may also suffer the problem of limited generalization ability. In addition, our model has an additional alignment module to learn the relationship between object candidates and grasp candidates, while MASK-GD cannot. Compared with other grasp detection methods, the improvement of our SOGD is more significant.

**Table 2:** Grasp detection results on the Jacquard Dataset.

**Methods**	**Accuracy/%**	**Input**
Zhou et al. (2018)	91.8	RGB
Zhang et al. (2019)	90.4	RGB
Dong et al. (2021)	**97.1**	RGB
Zhou et al. (2018)	92.8	RG-D
Zhang et al. (2019)	93.6	RG-D
Dong et al. (2021)	**97.6**	RG-D
SOGD (ours)	**99.0**	RGB-D

The best performance of methods within a same kind of input is shown as bold.

**Figure 6:**
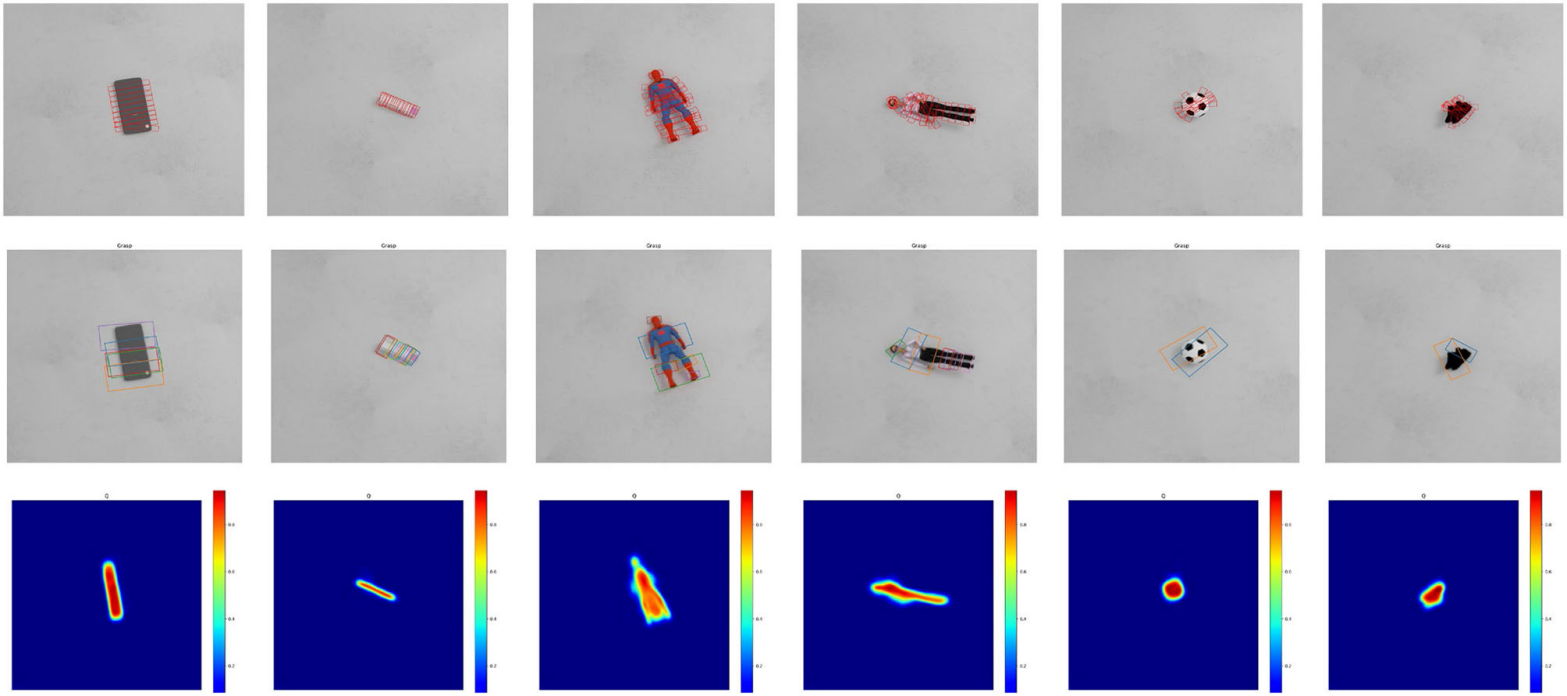
Grasp detection results of our SOGD on the Jacquard Dataset. **Top** line is ground truth annotation; **middle** line is the prediction of our SOGD; and **bottom** line is the estimated grasp quality of the corresponding detection.

Figure 6 shows some typical grasp detection results of our SOGD on this dataset. Both the detected grasp configurations and the quality maps are visualized to give a better understanding of the results. From the figure, it can be seen that our SOGD can well detect grasp candidates and outputs a reasonable quality map on this dataset.

### 4.3. Object detection results

**Figure 7:**
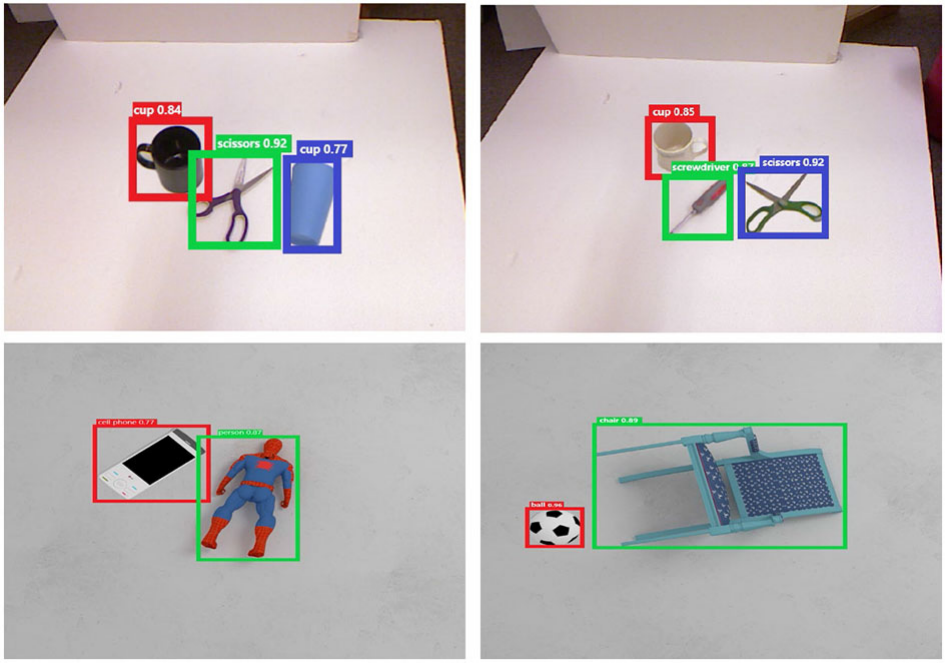
Typical object detection results.

For object detection evaluation, our SOGD model is trained and tested on the two datasets mentioned earlier. We use the Labelme tools from MIT to manually annotate bounding boxes and class labels on the Cornell Dataset. To prevent overfitting, the pre-trained parameters of ResNet-50 are fixed for the backbone and several data augmentation strategies are involved, such as rotation, translation, flip, random crop, and illumination change. Typical experimental results are shown in Figure 7. From the figure, it can be seen that after removing the background boundaries foreground objects are much easier to detect, releasing the burden of the object detection branch and resulting in more accurate bounding box predictions. In our experiments, though we pay more attention to the prediction accuracy of where the object is, the classification confidence of our SOGD is almost above 0.75 on the two datasets. The above-mentioned observations show that our SOGD model has a good performance in detecting objects from a cluttered scene, especially in identifying the boundaries of the objects.

### 4.4. Discussion on background removal

From the results in Tables 1, 2, it has already been seen that our SOGD has superior performance than existing methods without background removal (Zhang et al., 2019; Kumra et al., 2020; Yu et al., 2022b). But to investigate how much the background removal contributes to this performance boost, we conduct an ablation study on this pre-processing. The Jacquard Dataset is used in this experiment since it provides a ground truth mask for foreground target objects. With this mask, we generate two additional types of images from the original dataset to test the performance of our SOGD on it. The first one is to filter out backgrounds with the ground truth mask. The second one is to fill the background with a cluttered background. To achieve this, we download a number of images from the Internet as the background image datasets and randomly choose one to replace the background images from the Jacquard Dataset.

**Figure 8:**
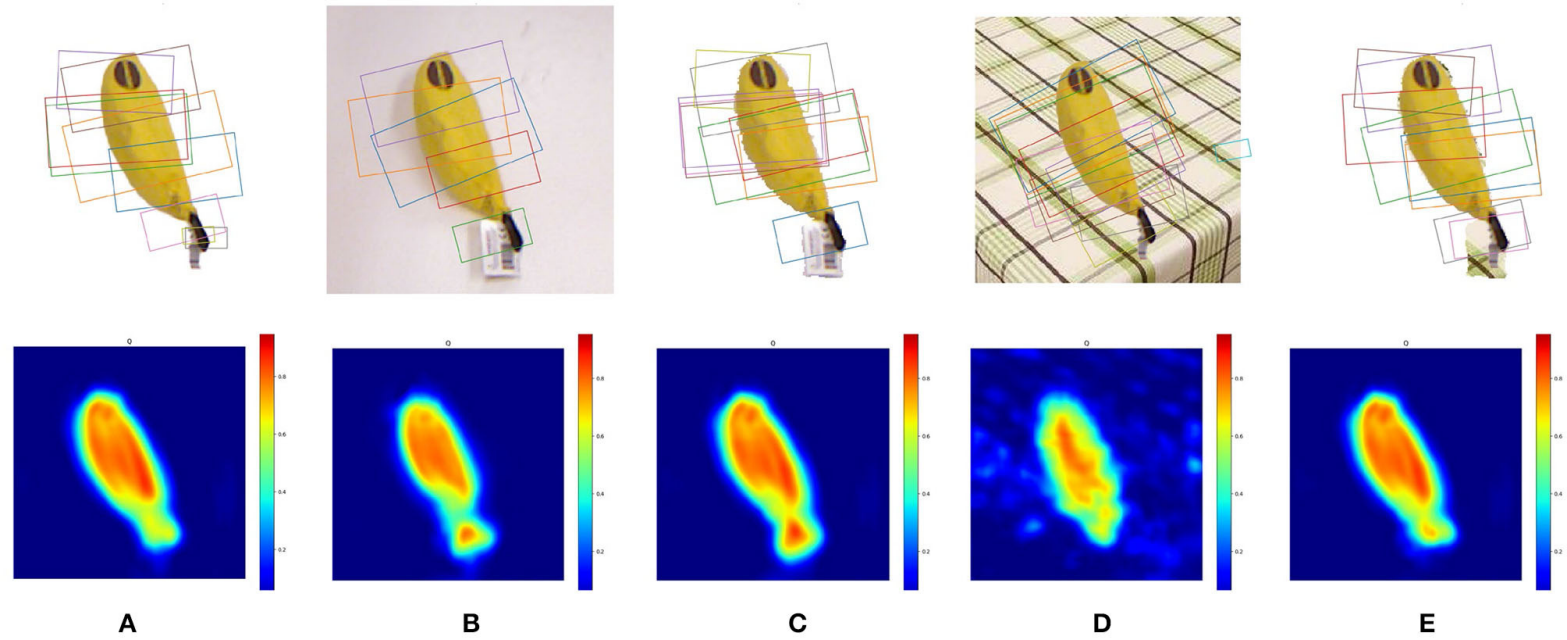
Grasp detection results on images with various kinds of backgrounds. **(A)** SOGD without background removal (BR) on images without background. **(B)** SOGD without BR on original images from Jacquard Dataset. **(C)** SOGD with BR on the original images. **(D)** SOGD without BR on images with the background being replaced. **(E)** SOGD with BR on images with the background being replaced. From left to right the background of the image becomes more cluttered.

We provide a comparison among five types of grasp detection configurations: (1) SOGD without background removal on images with background filtered out, (2) SOGD without background removal on the original image, (3) SOGD with background removal on the original image, (4) SOGD without background removal on images with background being replaced, and (5) SOGD with background removal on images with background being replaced. Typical results are shown in Figure 8. The background of the scene in Figure 8 becomes more cluttered from left to right. From the figure, we observe that the predicted grasp configurations will be much better when the background is removed from the image.

## 5. Conclusion and future study

This study is focused on the problem of grasp detection from an RGB-D image. Unlike previous methods, we solve this problem by simultaneously detecting the target object and the corresponding grasp configurations. This is motivated by the fact that when grabbing an object, we humans first identify where the object is and then make a decision on which part of the object to grab. To this end, a novel neural network SOGD together with its learning method is proposed. In SOGD, object and grasp configurations are first detected by two separate branches, and then the relationship between object candidates and grasp configurations is learned by an alignment module. The best grasp configuration is predicted according to the grasp score and its correspondence to the target object. Our method is tested on two publicly available datasets. A series of experiments are conducted and both qualitative and quantitative experimental results are presented. The results demonstrate the validity and practicability of our method.

To deal with grabbing in a cluttered scene, a pre-processing for background removal is designed. Unlike previous methods where background removal is treated as an image segmentation problem, we propose to leverage the prior knowledge that objects to be grabbed are often placed on a 3D plane. Therefore, we adopt a RANSAC-based plane fitting method to detect the largest 3D plane in the scene. All pixels laid in or under the plane are considered background. The experimental results show that our strategy makes grasp detection more robust in cluttered environments.

The stacked scene is not considered in this research. In daily life cases, it is common that objects to be grabbed are laid on top of each other. This is more challenging for the grasp detection method because it has to figure out the execution order of the predicted grasp configurations. This is an interesting topic for our future study. In addition, the kind of object for model training is limited. It has to face a large number of unknown objects when the learned model is deployed to real devices. It is interesting to extend our model with the life-long learning ability after deployment. We will explain it in our future study.

## Data availability statement

The original contributions presented in the study are included in the article/supplementary material, further inquiries can be directed to the corresponding author.

## Author contributions

YuaL contributed the main ideas and designed the algorithm. YuhL organized the database. LX performed the statistical analysis. YZ wrote the first draft of the manuscript. YZ, LX, YuhL, and YuaL wrote sections of the manuscript. All authors contributed to manuscript revision, read, and approved the submitted version.
